# C2 Synchondrosal Injuries: A Case Report and Anatomic Review

**DOI:** 10.7759/cureus.47649

**Published:** 2023-10-25

**Authors:** Chidi Nwachukwu, Wen Wang, Erik Soule, Amir Pirmoazen, Peter Fiester

**Affiliations:** 1 Radiology, University of Florida College of Medicine – Jacksonville, Jacksonville, USA

**Keywords:** axis fracture, craniocervical junction, magnetic resonance imaging, cervical trauma, synchondrosis

## Abstract

Developmental succession in the pediatric patient requires special consideration in post-traumatic assessment. An understanding of the sequential development of this region and common patterns of injury can provide an accurate initial assessment before proceeding to further management and prognostic evaluation. Primarily, this article focuses on the synchondrosal development of C2 and its role in the craniocervical junction, as well as its common patterns of injury. This article presents two sample cases and offers a review of treatment options with added prognostic factors.

## Introduction

Anatomic development predisposes the pediatric population to a unique array of injuries. The most common site of cervical spine injury is the craniocervical junction, which incorporates lax ligamentous attachments between the odontoid process, the atlas, and the occiput [1,2]. Sequential spinal ossification and laxity of supporting structures cause the cervical spine, specifically the craniocervical junction, to act as one of the most common sites of traumatic pediatric injury.

## Case presentation

Case 1

**Figure 1:**
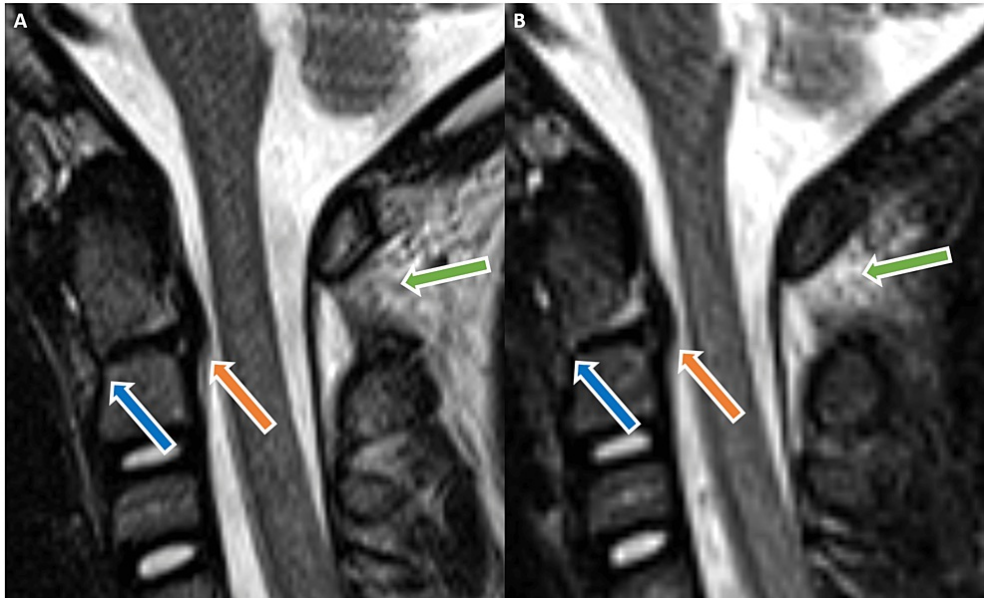
Sagittal T2-weighted (A) and STIR (B) MR imaging of the cervical spine demonstrates anterior subluxation of the dens with surrounding fluid (blue arrows). There is mild indentation of the ventral thecal sac secondary to truncated posterior longitudinal ligament (orange arrows). Widening of the posterior atlantoaxial space with associated hyperintensity is also noted (green arrows). STIR - short tau inversion recovery, MR - magnetic resonance

A five-year-old male presented after a high-speed motor vehicle collision complaining of right clavicular and chest pain. On examination, cervical spinal tenderness was present. No localized tenderness involving the thoracic or lumbar spine was reported. Computed tomography (CT) of the cervical spine was significant for anterior angulation of the dens with resultant increase in the basion-dens interval. Widening between the C1 and C2 spinous processes as well as atlantooccipital widening were also present. Subsequent magnetic resonance imaging (MRI) confirmed odontocentral synchondrosis injury with surrounding soft tissue abnormalities, including posterior longitudinal ligament irregularity and C1-C2 interspinous ligament injury (Figure 1).

The patient was admitted to the pediatric intensive care unit (PICU) and pediatric neurosurgery was consulted for further management. Upon assessment by pediatric neurosurgery, halo immobilization was initiated and implemented for approximately three months, after which the patient was transitioned to a cervical collar. Serial plain radiograph and CT examinations were performed to document appropriate callous formation and appropriate anatomic alignment. During long-term outpatient follow-up, patient complained of headache and residual pain at the sites of halo pin insertion. Repeat imaging evaluations indicated sinusitis as the likely etiology and demonstrated appropriate healing at the insertion sites.

Case 2

**Figure 2:**
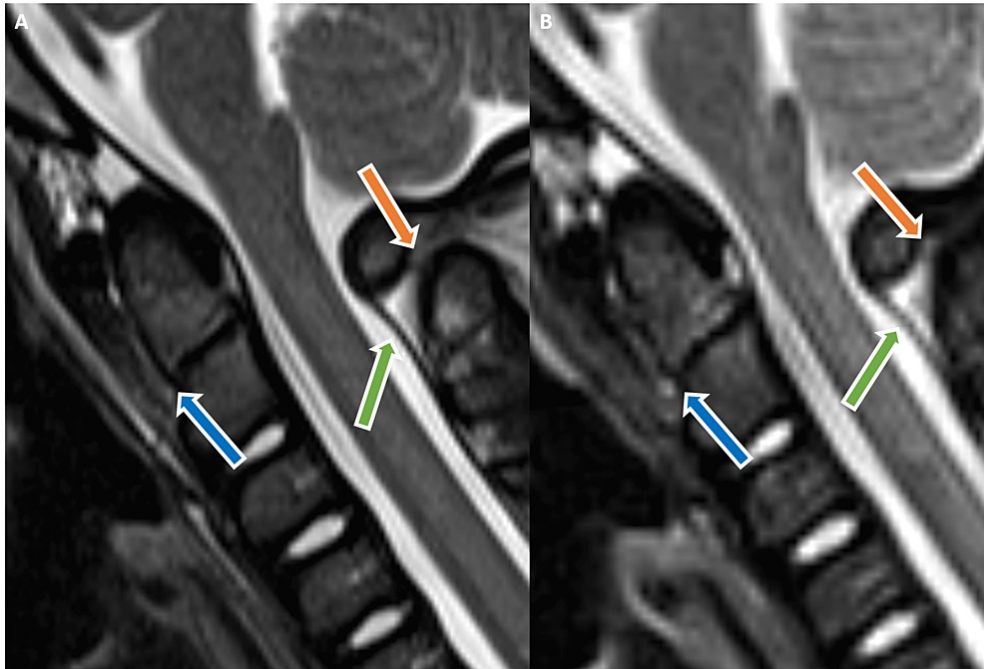
Sagittal T2-weighted (A) and STIR (B) MR images show abnormal hyperintensity with associated anterior tilting of the dens (blue arrows) consistent with odontocentral synchondrosal injury. Associated craniocervical junction posterior element injury involving the posterior atlantoaxial ligament (green arrows) and C1-C2 interspinous ligament (orange arrows) hyperintensity. STIR - short tau inversion recovery, MR - magnetic resonance

A five-year-old female was transferred to the emergency department after falling during play. She was reportedly doing flips on a couch and fell on the back of her neck, impacting a wooden portion of furniture. First responders at the scene implemented cervical immobilization for reported neck pain. On physical examination, pain with motion was present without cervical spine tenderness to palpation. Range of motion was also limited secondary to pain. Evaluation with CT of the cervical spine demonstrated mild anterior subluxation of the dens and C1-C2 interspinous widening, as well as atlantooccipital widening. A subsequent MRI confirmed odontocentral synchondrosal injury with evident posterior element injury (Figure 2).

The patient was admitted to the PICU, where regular neurological examinations were performed in addition to continued immobilization. Pediatric neurosurgery was subsequently consulted. Regular outpatient follow-up visits and continued faithful cervical immobilization via Miami J Collar were performed. On one-month follow-up visit, the patient reported continued neck pain that limited her range of motion, which prompted repeat follow-up imaging as well as prompt continued clinical follow-up. However, the patient was overall lost to follow-up in regard to the initial presenting facility’s pediatric neurosurgery clinic.

## Discussion

**Figure 3:**
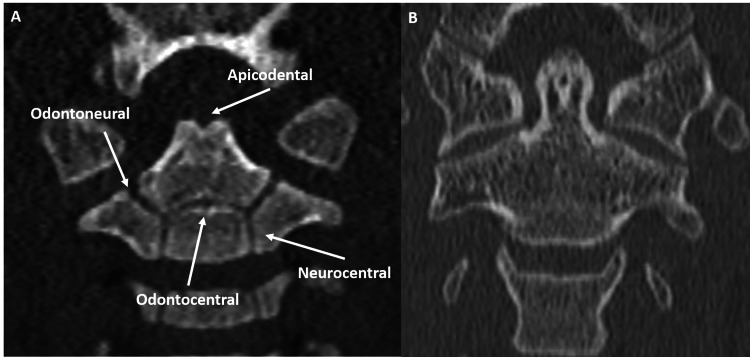
Coronal CT image demonstrates a normal developing C2 vertebra in a two-year-old (A). The os odontoideum has not yet ossified, but the apicodental synchondrosis is appreciable. The odontocentral is the other midline synchondrosis. The remaining odontoneural and neurocentral synchondroses are symmetric. Coronal CT image depicts adult C2 vertebra (B) on coronal axis for comparison. Note apical ossification and complete vertebral fusion. CT - computed tomography

Formation of the axis occurs via the sequential development and fusion of five ossification centers, which include the body, the odontoid process, the os odontoideum, and two neural arches. The os odontoideum, which also may be referred to as the os terminale or chondrum terminale, later fuses with the odontoid process ossification center to become the tip of the odontoid. Between the os odontoideum and the odontoid process ossification centers is the apicodental synchondrosis. The odontoid process also directly fuses with the body and two remaining neural arches ossification centers. Between the odontoid process and the body is the odontocentral synchondrosis. The odontoid process interface with bilateral neural arches is called odontoneural synchondroses (Figure 3).

The C2 body ossification center has its own points of fusion with bilateral neural arches along the neurocentral synchondroses. Of note, the odontoid ossification center normally presents at birth, which results from the fusion of two ossification centers for the odontoid process [3,4].

The main joint articulations of the craniocervical junction are the atlantooccipital and the atlantoaxial, which are key to cervical spine mobility. The mobility displayed by these articulations is a direct reflection of the ligamentous laxity at this level, and much of the force translated through extension and flexion is mitigated by the atlantoaxial articulation [5]. The tectorial membrane is a supra-odontoid extension of the posterior longitudinal ligament that cranially merges with the dura, allowing it to act as a key component of the craniocervical junction’s complex fibrous support. Although the tectorial membrane extends cranially to become indistinguishable from the dura, it is not fully attached to the skull in pediatric patients [6]. At the level of the atlantoaxial articulation, the transverse and alar ligaments provide the most stabilization, which allows significant injury when these ligaments are disrupted. The transverse ligament is a strong, thick ligament that attaches to the medial aspects of the C1 lateral masses, which constrains the odontoid process against the posterior aspect of the anterior C1 arch. The epidural fat pad and the tectorial membrane, as it transitions to the cranial dura, are located posterior to the transverse ligament. The alar ligament is a craniocaudally oriented fibrous structure that attaches the posterior odontoid process to the skull base with attachment ranging from the medial occipital condyles to the foramen magnum. These ligamentous attachments limit extensive flexion, extension, and rotation. Despite providing support and limiting hypermobility, inherent ligamentous laxity in children may mimic injury, causing radiologic patterns such as atlantoaxial pseudosubluxation [7].

As the ossification centers of the pediatric spine mature, they are biomechanically supported by lax neck musculature and relatively malleable ligaments, which results in spinous hypermobility. For pediatric patients, the head acts as a heavy loading force for the relatively weak cervical spine, allowing the cervical spine to be particularly vulnerable to hyperflexion and hyperextension injuries when exposed to forceful acceleration and deceleration. The most common mechanisms of injury occur secondary to motor vehicle accidents, which routinely involve abrupt acceleration and deceleration. This is particularly applicable to the C2 synchondroses, which are prone to traumatic dislocation prior to fusion and are particularly vulnerable secondary to the laxity of the surrounding soft tissues [8]. Proper assessment of C2 is a key skill in the post-traumatic management of pediatric patients.

Assessment of multiple ossification centers at the C2 vertebrae may cause uncertainty in regard to possible C2 injury by mimicking fracture if normal developmental patterns are not recognized. Normally, there are four ossification centers present at birth. An additional ossification center deemed as os odontoideum or os terminale appears between the ages of three and six, fusing via the apicodental synchondrosis by age 12 to form the odontoid apex. The ossification center may remain unfused, causing an appearance that may mimic odontoid fracture, though characteristically well-corticated margins are present. Odontoid fractures remain one of the most common fractures involving the pediatric cervical spine, and recognition of synchondrosal variation assists in their accurate diagnosis. If there is incomplete apicodental synchondrosal fusion, then the developing odontoid process is also particularly vulnerable to subluxation from hyperflexion.

On CT, normal synchondroses appear as hypoattenuating lines separating well-corticated ossification centers. On MRI, they appear as linear T2 hypointensities separating expected ossification centers. These synchondrosal articulations may dislocate or fracture into the four most commonly described fracture patterns, which in some cases may resemble adult correlates, such as variable odontoid fractures. On MRI, the visualization of abnormal T2 hyperintensity involving synchondrosis can be an indicator of an underlying injury.

**Figure 4:**
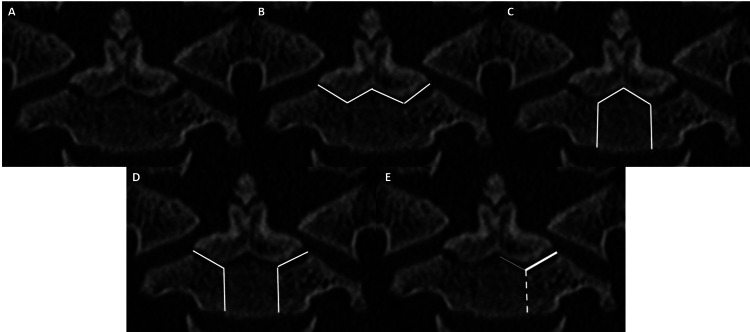
Coronal CT (A) shows calcified os terminale with unfused apicodental synchondrosis. Remaining synchondroses also incompletely fused, as expected for age. Coronal CT image (B) demonstrates a theoretical type 1 fracture through bilateral ondontoneural and midline odontocentral synchondroses (white line). Coronal CT image (C) demonstrates a theoretical type 2F fracture through bilateral neurocentral and midline odontocentral synchondroses (white line). Coronal CT image (D) demonstrates theoretical type 3 fracture through bilateral neurocentral and odontoneural synchondroses (white lines). Coronal CT image (E) demonstrates a theoretical type 4 fracture through either odontoneural synchondrosis with or without adjacent injury to bordering odontocentral or neurocentral synchondrosis. Provided image depicts left odontoneural (thick, solid line) and possible injury to adjacent odontocentral (thin, solid line) or neurocentral (dashed line) synchondroses. CT - computed tomography

During radiologic assessment, it is important to recognize the common injury patterns and understand the normal developmental anatomy. The synchondrosal fracture patterns, most avidly described by Rusin et al., fall into four types: type I (fracture through both odontoneural and odontocentral), type II (fracture through both neurocentral and the odontocentral), type III (fracture through both odontoneural and both neurocentral), and type IV (fracture through an odontoneural with or without a portion of the bordering odontocentral or neurocentral). The type IV pattern is an incomplete fracture more frequently associated with minor ligamentous injury. In a pediatric patient with C2 synchondrosal injury, further clarification can be made toward the fracture pattern based on the severity of injury (Figure 4).

The type I-III fracture patterns can be further subtyped based on degree of displacement from a to d, where a refers to 0-10% dislocation, b to 10-100%, c to >100%, and d to distraction [9-11]. Presence of more severe injury patterns is highly associated with soft tissue abnormalities, which may be visible on MRI. Visualization of odontoid inclusion in the mechanism of injury may be associated with retroclival epidural hematoma, which is a sign of tectorial membrane injury.

The management of pediatric craniocervical injuries slightly differs from that of adult counterparts. Odontoid subluxation at the level of the odontocentral synchondrosis or odontoid epiphysiolysis is most commonly managed with halo immobilization to allow re-articulation and fusion, which can take from 10 to 18 weeks. Though halo immobilization may commonly be utilized, other manners of closed reduction and external immobilization, such as with a Minerva jacket, have also proven successful. Surgical internal fixation can be performed as an initial treatment or following unsuccessful fusion or alignment. On the other hand, more studies recommend surgical internal fixation, rather than halo immobilization, for management of epiphysiolysis involving other C2 synchondroses. Surgical treatment of these injuries includes operative wire cerclage, anterior odontoid screw fixation, anterior or posterior screw fixation, and traction device implementation. Halo immobilization may be used in patients lacking signs of spinal cord injury, which is more common in types a and c. In patients undergoing pin fixation, insertion sites should be assessed for signs of infection. Incomplete type IV fractures may commonly be treated with conservative cervical collar placement.

## Conclusions

The pediatric population is more susceptible to traumatic C2 vertebral injury secondary to age-dependent anatomic laxity exemplified by the piecewise development of the C2 vertebra. A developmental understanding of the composition of C2 is crucial in abating misinterpretation of injury. Recognition of the common patterns of C2 injury involving the synchondroses can lead to more accurate reporting of acute traumatic injuries. Traumatic injury to the C2 arch can be categorized according to common fracture patterns with graded severity. These fracture patterns can predict underlying spinal injury and affect management. Therefore, classifying different C2 fracture patterns in the pediatric population is essential for predicting the outcome and planning the treatment.
